# Human-centered design process and solutions to promote malaria testing and treatment seeking behavior in Guyana hinterlands

**DOI:** 10.1186/s12889-021-12297-0

**Published:** 2021-12-15

**Authors:** Shirley D. Yan, Joann Simpson, Lyndsey Mitchum, Jennifer Orkis, TrishAnn Davis, Sean Wilson, Neil Trotman, Helen Imhoff, Horace Cox, Gabrielle Hunter, Bolanle Olapeju, Camille Adams, J. Douglas Storey

**Affiliations:** 1 Johns Hopkins Center for Communication Programs, 111 Market Place, Suite, Baltimore, MD 310 USA; 2 Noora Health, San Francisco, California USA; 3 Breakthrough ACTION Guyana, XX Barrack St., Georgetown, Guyana; 4 Vector Control Services, Ministry of Health, Middle Street, Georgetown, Guyana

**Keywords:** Guyana, Malaria, Human-centered design

## Abstract

**Background:**

Malaria is a persistent public health challenge among miners and other hard-to-reach populations in Guyana’s hinterland, specifically in Regions 1, 7, 8, and 9. Despite an overall decrease in malaria prevalence throughout Guyana, it remains common among mining populations whose work conditions both contribute toward malaria transmission and make it difficult to seek timely, Ministry of Health (MoH) approved malaria testing and treatment services. In an effort to develop innovative approaches to address this public health challenge, an interdisciplinary team of public health professionals, designers, and mining organizations collaborated using a human-centered design (HCD) process facilitated by the USAID-funded Breakthrough ACTION Guyana project in partnership with the MoH.

**Methods:**

This paper describes two phases: [1] Define and [2] Design & Test. In the Define phase, following a literature review, we conducted 108 qualitative interviews with miners, camp managers, trained malaria testers, health workers, and other key stakeholders to understand experiences and challenges when seeking malaria testing and treatment services. These interviews were synthesized into 11 insights on issues such as risk perception, malaria knowledge, preventive behaviors, traditional and self-treatment, adherence to the correct treatment, testing, and coordination and communication gaps. From these insights, during the Design & Test phase, we developed 33 “How might we…?” questions which led to 792 ideas, of which eight emergent concepts were prototyped and refined in the field with 145 miners, camp managers, and stakeholders.

**Results:**

The five final prototypes included: “Little Mosquito, Big Problem” social behavior change campaign; rapid counseling cards; branded malaria testing and treatment services; innovations in treatment adherence; and a participants, content, and logistics approach.

**Conclusion:**

When applying HCD to public health issues, there are both opportunities and challenges to reconcile gaps that may exist between the two disciplines. However, HCD provides additional tools and mindsets to generatively work with migrant and mobile mining communities to encourage malaria testing and treatment services.

## Background

Malaria is a major public health problem in Guyana, mainly in the hinterland—Regions 1, 7, 8, and 9. Though considerable progress has been made, trends in the case data demonstrate challenges for malaria elimination. The Guyana National Malaria Programme (NMP) reported in its Strategic Plan 2020–2025 a 20% reduction in the number of new cases between 1996 and 2012, although the period was characterized by intermittent peaks in cases. However, in recent years (2015–2019), malaria incidence has been on the rise with a noticeable reduction of 21.9% in testing rates over the same period. The majority of malaria cases in Guyana are caused by *Plasmodium vivax* (64%)*,* although *Plasmodium falciparum* (30%)*,* and *Plasmodium malariae* (0.2%) are also present*.* Human activity in tropical rainforest settings, such as gold mining, creates favorable habitats for mosquito breeding, which increases risk of mine workers’ exposure to malaria [1]. Gold mining in the hinterland is significantly associated with malaria as it favors the proliferation of malaria vectors through deforestation [2]. According to the Guyana Population and Housing Census Report 2012, gold mining is the main economic activity in Regions 1, 7, 8, and 9, which in turn accounts for 85–95% of the total malaria cases in the country [3]. Further, the National Malaria Program Strategic Plan 2020–2025, reported that peaks in malaria cases may be associated with an increase in international gold prices and consequent large-scale increases in mining activities [4]. Many economic migrants typically move from coastal areas to the hinterland to engage in mining and related activities. To reduce malaria prevalence, the Ministry of Health (MoH), through its Vector Control Services (VCS) department has increased malaria diagnosis and treatment targets and vector control interventions like long lasting insecticide treated nets (LLINs); social and behavior change (SBC) efforts; indoor residual spraying upon response to outbreaks; and surveillance of outbreaks, cases, drug resistance, and vectors.

Supported by the Government of Guyana and Global Fund to Fight AIDS Tuberculosis and Malaria (GFATM) funding, diagnosis and treatment are free of charge in public health facilities to promote accurate malaria testing and treatment. Although microscopy remains the gold standard for malaria diagnosis, rapid diagnostic tests (RDTs) are increasingly used in areas or settings where access to microscopy is limited. To increase access to diagnosis and treatment services, VCS trains volunteers who work in mining, logging, and other remote communities to test for uncomplicated malaria using RDTs and to treat all positive cases (except for pregnant women, children, and severe cases). The volunteers identify symptomatic cases of malaria through passive case detection; identify and refer severe cases to the closest health facility and refer children and pregnant women to a health facility as well. The volunteers recruited for this community case management program are generally stable workers in the mining community such as cooks, camp managers, security guards, and shopkeepers. While there is no tangible incentive provided to the volunteers, they are provided with RDT test kits and treatment once they complete the MoH training.

In Guyana, *Plasmodium falciparum* is treated with a three-day course of artemether-lumefantrine and a stat dose of primaquine whereas *Plasmodium vivax* is treated with a three-day course of chloroquine and a fourteen-day course of low dose primaquine. These effective treatment for malaria are available through official MoH sources, such as the trained volunteer testers or at health facilities. However, sub-standard, poor quality, and unauthorized medicines are marketed for malaria through informal sources. Often, these drugs contain monotherapies for malaria which threaten to promote antimalarial resistance. The remoteness of the mining and logging camps makes it difficult for the public and private sectors to offer services. In the absence of this volunteer programme, some mining camps purchase antimalarial medicines and encourage miners to self-medicate. Even where MoH testing and treatment is novel there are knowledge, attitudes and risk perceptions of the target population that act as barriers to access. Poor compliance with the full recommended treatment regimen for malaria constitutes a significant risk for the emergence of malaria parasites resistant to antimalarial drugs [2]. Preliminary results of a 2018–19 artemether-lumefantrine therapeutic efficacy study did not reveal links to in vivo resistance in Guyana; however, caution must be exercised to prevent emergent resistance in Guyana.

The USAID-funded Breakthrough ACTION Guyana project (2017–2021) partners with VCS, the Public Relations/Health Promotion Unit (PR/HPU) of the MoH, the Pan American Health Organization (PAHO), the Word Health Organization (WHO), and GFATM to improve malaria outcomes among gold mining populations by promoting prompt malaria testing and treatment-seeking behavior. The PR/HPU in the MoH manages public-facing media inquiries for the Ministry and supports health communication activities among the various departments. Breakthrough ACTION Guyana collaborates with these key partners to:

1. Design and implement targeted, innovative, and effective solutions to high-priority social and behavioral challenges
2. Increase capacity of Guyanese institutions to coordinate, design, implement, and evaluate high-quality social behavior change (SBC) programs

Breakthrough ACTION Guyana uses the SBC Flow Chart to achieve these two outcomes. The SBC Flow Chart, developed under USAID’s global flagship SBC project, Breakthrough ACTION, draws inspiration from several fields, including human-centered design (HCD), audience segmentation, and behavioral economics. Over the last decade, global health projects and organizations have increasingly used HCD to understand, ideate, and address difficult to solve health problems [5]. The HCD approach encompasses a mix of creative processes, mindsets, and skills that integrate different types of design to solve problems [6]. HCD has been applied and used in global health issues such as tuberculosis, HIV, chronic disease management, infectious disease, sanitation and hand hygiene, family planning, and infant mortality [5]. Despite the opportunities and strengths HCD brings to public health challenges, there are also challenges and weaknesses with falsifiability, replicability, and questions as to whether designed solutions would work across scale [5].

Within the malaria field, MacDonald and Putzer argue to increasingly use HCD approaches to improve malaria outcomes within the context of LLIN use, because “people impacted by malaria will be enabled to guide us according to their priorities, how they live, and what is important to them” [7]. Previous uses of HCD to develop strategies to combat malaria have included an analysis of consumer behaviors for insecticide treated bed nets in Ghana [8]; and use of HCD methodologies to engage and understand community perspectives and priorities for malaria prevention strategies in Zambia [9]. Use of HCD techniques and processes within malaria has not been documented in literature, especially for malaria testing and treatment services. In the work of Breakthrough ACTION Guyana, HCD was used to guide the development of solutions to encourage miners to seek testing and treatment services.

This article aims to describe how the project team used HCD, with a focus on the Define and Design & Test phases of the SBC Flow Chart, to develop interventions to increase use of malaria testing and treatment services by gold miners. If their access to accurate malaria testing and treatment services increases, miners are more likely to cure their malaria. As a result, the chance for drug resistance malaria and malaria transmission among hard-to-reach populations reduces.

## Methods

**Fig. 1:**
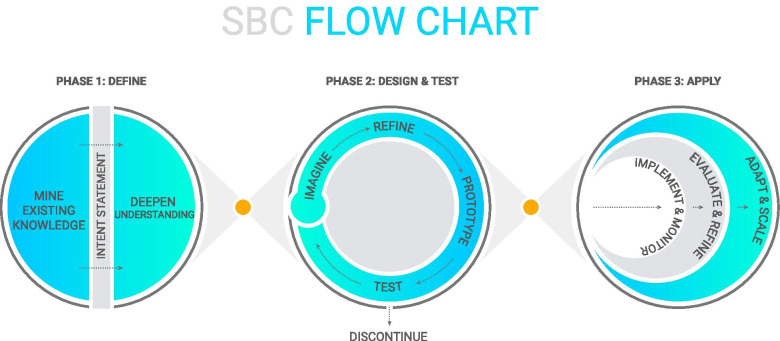
Social behavior change flow chart

The SBC Flow Chart (Fig. 1) is an integrated, trans-disciplinary approach that aims to gain a deep understanding of the full scope of the problem (Phase I: Define), translate problems into solutions through iterative cycles of design (Phase II: Design & Test), and implement programs more nimbly, using real-time monitoring to adapt and course-correct (Phase III: Apply). Project teams leverage different disciplines in the application of the SBC Flow Chart based on the specific needs of the project. Breakthrough ACTION Guyana’s approach relied heavily on HCD methodologies in order to put the audience at the center throughout, generate a wide range of ideas not limited only to social and behavior change communication (SBCC), and co-create and rapidly iterate low-fidelity prototypes.

HCD shares similar inspiration from social science disciplines such as anthropology, sociology, psychology, and human factors design. While HCD offers an opportunity to rapidly and directly synthesize findings from field data collection, there are some deviations from typical socio-behavioral science approaches to problem solving. Given the exploratory nature of HCD work, especially in the Define phase where formative research occurs, there tends to be a lack of hypothesis-driven science research and a lack of clear documentation as to what happened and the rationale behind each decision, though this varies between projects [5]. During the Design & Test phase, in which users test solutions that further iterate, the process tends to have less focus on an auditable and replicable process compared to projects led by socio-behavioral sciences [10]. Given these challenges, the authors employed health research reporting guidelines for HCD projects to encourage replicability and proper dissemination for this manuscript [11]. Throughout the project, the team documented the process using reflection sessions after it completed each phase.

This article highlights the implementation of two of the three SBC Flow Chart phases—Define and Design & Test—in the context of malaria reduction among gold miners in Guyana.

The Johns Hopkins University School of Public Health Institutional Review Board classified this activity as a non-research activity and within the domain of public health practice. The Ministry of Public Health Guyana, Ethical Review Committee gave ethical approval.

For both the Define and Design & Test phases, two teams of seven people—each consisting of experienced social scientists from Breakthrough ACTION, the MoH VCS and PR/HPU, and design specialists (e.g., visual designers, design strategists)—conducted field research in Regions 7 and 8.

Interviews and focus group discussions (FGDs) were held in areas accessible and close to Puruni (Region 7) and Mahdia (Region 8). Details of the field site visit are documented further in a previously published article [12]. For each phase and stage of the implementation of the SBC Flow Chart, the VCS (central and regional) and PR/HPU were integral participants of the workshops and members of the research teams through which their capacity was built in a ‘learn-by-doing’ approach.

Table 1 outlines stages, descriptions, project components, and outputs for the phases of the SBC Flow Chart. The methods followed have been documented in a previous non-peer reviewed report and are mentioned here again for clarity [13].

**Table 1:** Outline of stages, descriptions, project components, and outputs for the SBC Flow Chart phases

Stage and Date	Description	Project Components	Outputs
Define Phase: Mine Existing Knowledge July–December 2018	This stage establishes a current understanding of the challenge using existing qualitative and quantitative findings from published literature or secondary data analysis.	Literature review	Literature review report
Define Phase: Intent October 23, 2018	In this stage, the objective is to determine the changes that the co-design and research teams would like to see after the implementation of an intervention, namely, in the short, medium and long terms. In this stage, likened to a navigator setting the direction, multiple and diverse perspectives are considered until consensus is reached by the participants.	One-day intent workshop with key stakeholders in Georgetown	Intent statement
Define Phase: Deepen Understanding October 24–November 8, 2018	In the ***Deepen Understanding*** stage, answers are sought for key questions, such as: What do people want and need? What are their problems and challenges? Informal and semi-structured interviews and in-situ observations are used to answer these questions. The aim is to base design decisions on a deep understanding of people’s lived experiences.	Two-day capacity strengthening workshop, five-day field-based qualitative interviews, one-day insights harvesting workshop, one-day insights validation workshop	Insights from field research and design artifacts
Design & Test Phase Stage I: Imagine and Refine March 12–13, 2019	The Design & Test phase generates many ideas and like an architect trying to stay focused, the questions that are constantly asked are, “What ideas could change the way things are? Which should we run with?” Primarily, this stage allows for the embracing of new possibilities, challenges and futures.	Two-day imagine workshop in Georgetown to generate, develop, and prioritize ideas	Emergent solution themes
Design & Test Phase Stage II: Prototype and Test March 14–29, 2019	In the ***Prototype*** stage, key ideas are grouped, and theme and low-fidelity solutions are generated and tested with the beneficiaries. This stage is likened to a true experimenter who always tries, fails, and learns. The motivation is, “let’s improve it.” This stage can be repeated several times with increasing levels of fidelity and refinement.	Two-day prototype workshop in Guyana to build prototypes, five days of field testing of prototypes and emergent ideas, one day of presentations, one day of debrief	Prototypes
Apply Phase: Implementation June 2019-	The final step in the process is to ***Launch or Implement*** higher fidelity versions of the prototypes that emerged as the most feasible and desirable during the Prototype stage. In other words, “it/they are looking good, let’s get it/them out there!”	Not implemented during this timeframe	Implemented solutions at scale

### Define phase

The Define phase included a comprehensive review of the literature, an Intent Workshop, and the deepening understanding component (field-based research).

#### Literature review

To gain a further understanding of the malaria situation in Guyana and its surrounding countries, a review of existing literature was conducted to identify malaria knowledge, attitudes, and practices; behavioral barriers; and gaps in information, social, and cultural context of mining communities in the priority regions. An initial search was conducted using PubMed, EmBase, and Google Scholar for peer-reviewed journals on the aforementioned topics in Guyana and the Amazon Basin available in English. Country-level documents, project reports, and other grey literature were also collected for Guyana and surrounding countries. The search yielded limited documents and was therefore expanded to include other low- and middle-income countries not located in South America. A total of 40 relevant malaria-related documents were found: 24 specific to Guyana and 16 focused on other countries. The results of the literature review demonstrated that gaps in malaria knowledge around transmission, prevention, and treatment exist and there are opportunities to correct misunderstandings and improve behaviors. Literature review results confirmed information that was already known by the team regarding access to malaria testing and treatment services. It also highlighted additional topics to explore as part of future research.

#### Intent workshop

The Intent Workshop aimed for the core design and research teams to gain a high-level understanding of the malaria and mining context in Guyana and to agree upon the project purpose in the short, medium, and long term. The main output at the end of the workshop was a draft intent statement. Achieving this objective required the involvement of key stakeholders at the workshop. Twenty-four participants were drawn from the MoH, Ministry of Communities, Ministry of Indigenous People’s Affairs, mining organizations (e.g., The National Mining Syndicate; Guyana Women’s Miners Organization), Merundoi (nongovernmental organization that specializes in behavior change communication), PAHO/WHO, USAID, and Breakthrough ACTION.

During the one-day workshop in October 2018, participants deliberated over several key issues in small groups and in plenary. These included understanding the current state of malaria in Guyana and the desired future state. Presentations by the MoH, Breakthrough ACTION, and participants’ experiences working in the mining sector of Guyana aided the discussions and allowed for fruitful conclusions to such questions as: What does the current state mean for stakeholders? What will success look like for the project in the short, medium, and long-term?

#### Deepen understanding

In this stage, the project conducted field research and data processing. The results were used to harvest/validate insights and develop design artifacts.

Prior to the Deepen Understanding stage, Breakthrough ACTION Guyana conducted mobilization visits in both Regions 7 and 8. The purpose of these visits was to identify existing mining camps and meet with camp managers to inform them about the research. When meeting with the camp managers, researchers specifically inquired about the best time to speak with the miners. A schedule for the teams traveling to each region was developed so that interviewers would not show up at the mining camps unannounced, and at an inopportune time for the miners.

In this stage, research depends on the ability to conduct quasi-ethnographic, personalized, in-depth discussions with members of relevant stakeholder groups. The process uses interview techniques that are common in various forms of qualitative research but emphasize the importance of collecting personal narratives that reveal stakeholder experience and desired future states. The skills needed to conduct these kinds of interviews do not come naturally to most people; skilled qualitative interviewers often have years of training and experience. However, one mandate of the Breakthrough ACTION Guyana project was to build the capacity of local partners to carry out high-quality SBC programs. While some of the Guyanese counterparts had extensive experience as field supervisors and outreach workers, none had any training in qualitative research, hence the need for a capacity strengthening workshop. As part of the capacity strengthening workshop, the teams who would conduct discovery research in Regions 7 and 8 participated in sessions on the differences between qualitative research and other types of information gathering, the structure of an in-depth interview, the importance of a respondent-centered mindset, rapport building, mindful listening, the use of open-ended questioning techniques, probing, and research ethics. Participants then practiced conducting interviews with each other using the interview guides.

#### Field research

**Fig. 2:**
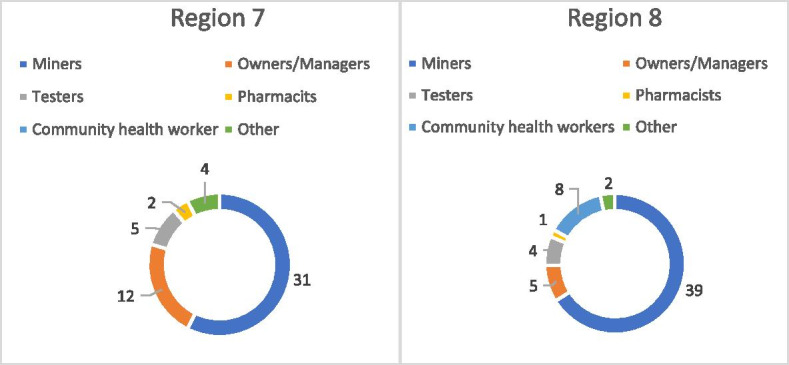
Summary of participants from qualitative fieldwork

Two research teams of seven people each, including staff from Breakthrough ACTION, central and regional VCS, and MoH PR/HPU who participated in the capacity strengthening workshop, conducted research activities during a one-week period in October 2018 at mining communities across Regions 7 and 8. In Region 7, research focused on two major communities and six mining camp areas, while in Region 8 the team focused on one major population center, four mining camp areas, and a rural health post. A total of 108 people (miners, camp managers, health testers, regional administration, media professionals, and hospital staff) from these communities were interviewed (Fig. 2).

The objective of the deepening understanding interviews and FGDs was to understand the experiences of each stakeholder group around malaria-related behaviors. Separate “lines of inquiry” (or interview guides) were developed for each stakeholder group so that conversations would be relevant to each context. For example, the lines of inquiry for miners focused on understanding a standard day of work in the mines, perceptions about personal health including preventing and coping with malaria, as well as their relationships with camp management and health services. For camp managers, the lines of inquiry focused on the context of the camp, their relationships with miners, the challenges malaria poses for their professional life and the business of mining, and their role in protecting the health of their workers. For the volunteer RDT testers, discussions focused on how they perceive their role as a tester, how they relate to and communicate with miners who come to them for services, attitudes and behaviors towards negative RDT results even with malaria-like symptoms, the challenges inherent in their work including reporting requirements, and their relationship to the health system that provides testing and treatment supplies, training, and supervision.

Interviews and FGDs were conducted in available spaces where some degree of privacy was possible at camps or in the larger communities. Each interviewer was supported by a note-taker/observer who also operated a handheld audio recorder. Prior to initiating each interview, the purpose of the activity was explained and consent was sought from all participants to conduct and record the interviews. Field teams took photos and recorded general observations of the field sites to document findings relevant to the lines of inquiry. At the end of each day, the interviewer and observer transferred their notes and observations onto a summary sheet, reviewing the recordings if necessary, and capturing the highlights of each conversation, including: the respondent’s personal motivations in daily life; challenges they face; things that give them satisfaction in their life; perceptions about malaria testing, treatment, and adherence; and any initial insights or interesting findings worth noting.

#### Data processing

At the end of the week, the research team in each region met for a daylong session to compile the findings across all of the interviews. First, the team transcribed highlights of the interviews onto colored Post-It™ notes, with one finding per note. Findings were color-coded by stakeholder groups. Findings could include quotes, paraphrased comments, observations, facts, or initial insights. The goal was to get information out of research notes and researchers’ heads and physically onto the wall where it could be processed collectively.

After extracting the findings, each research team worked together to group the information by sub-themes through an iterative process of clustering, discussing and comparing clusters, then reclustering until all findings were located in a larger map of themes and concepts. The two regional teams used slightly different approaches: Region 7 grouped findings irrespective of stakeholders (that is, combining similar or related findings across stakeholder groups), while Region 8 grouped findings and created theme clusters within stakeholder groups however, both teams generated similar sub-themes. Examples of sub-themes included such things as malaria knowledge, care-seeking behaviors, motivations to enter into mining, misconceptions about malaria, and other health concerns. A total of 92 themes and sub-themes were generated across the two regions. Each step of the clustering process was photographed in order to allow retrospective examination of how the clusters emerged and changed and to identify the final insights. The physical artifacts (flipcharts covered with clustered Post-It™ notes) were rolled up and transported back to Georgetown, where an Insights Validation Workshop was held with the broader stakeholder group from the initial Intent workshop including reresentatives from the MoH.

In Georgetown, each research team reviewed the other team’s data and collapsed common sub-themes into broader themes across regions. Some sub-themes remained specific to a stakeholder group while others were crosscutting. For example, all of the sub-themes on malaria knowledge, knowledge about malaria transmission, and malaria symptoms were collapsed into a broader “Malaria Knowledge” theme. Afterward, team members focused on one theme and individually reviewed the findings associated with each sub-theme to identify key ideas that were representative of the broader theme (e.g., a compelling quote from a stakeholder). Initially, the research team generated 14 themes. These themes were later consolidated into the 11 themes that correspond to the insights presented in this report [13].

#### Insights harvesting and validation

After finalizing themes, researchers (typically from two different regional research teams) worked in pairs to review findings and generate preliminary insights related to a particular theme. An insight has three distinct characteristics: [1] it comprises two or more disparate pieces of information [2] that combine to shed new light on a situation [3] in a way that suggests opportunities for action to collectively generate, develop, and prioritize ideas around how to improve malaria testing and treatment among miners in Guyana.

#### Development of design artifacts

After crafting and refining the insights, the Breakthrough ACTION Guyana team reviewed each insight individually to identify one or more “design opportunities.” These opportunities frame the specific challenges proposed in each insight and are presented in the form of a question that starts with, “How might we…?”

“How might we” (HMW) questions help a design team think about the opportunities and challenges implied by an insight in a constructive way. A well-crafted HMW question does not suggest only a single solution. It should be aspirational and encourage a broad range of answers that point toward potential solutions to a particular challenge. Moving into the subsequent Design & Test phase, the HMW questions provide a quick and effective way of translating insights into ideas, concepts, and solutions that can be prototyped, co-designed, pilot tested with stakeholders, and ultimately implemented.

The team created other design artifacts such as personas and journey maps for miners, camp managers, volunteer testers, and community health workers. Personas are key archetypal users that represent the needs, goals, values, and behaviors of larger groups of people [14]. In this case, they allow us to understand our target audiences in a real and human way. Personas allow us to make evidence-based decisions, which means that all persona information is derived directly from our fieldwork. Acting as stand-ins for real people, personas are tools that help guide design teams in asking the right questions, generating insights, and ultimately making decisions about the functionality of a solution.

Journey maps illustrate the experience pathway or “journey” of a persona from their individual perspective and allow us to highlight pain points (e.g. challenge, barriers, or friction) and opportunities for intervention [15, 16]. Journey maps tell the important stories of our personas in a way that places them within a broader ecosystem of interactions between people and systems; they help us to consider our personas within their unique context, rather than in isolation. Most journey maps include a timeline, opportunities for intervention, and elements of the persona such as pain points, thoughts, and feelings. Journey maps are useful during the Design & Test phase because they help us to keep the experiences and interactions that influence behavior at the forefront.

### Design & test

Stage I of the Design & Test phase included an Imagine Workshop which used HWM questions in the development of prototypes, based on the insights that emerged from field research during the Define Phase.

Stage II of the Design & Test phase included the testing of those prototypes and the decision-making process used to prioritize solutions.

#### Design & test Stage I: imagine and refine

##### Imagine workshop

The Imagine Workshop took place in Georgetown over four consecutive days in March 2019. Through a structured yet flexible approach, the objective of the workshop was to collectively generate, develop, and prioritize ideas around how to improve an effective malaria testing and treatment program in Guyana to increase use of MoH services.

Thirty-five participants attended the workshop from 10 distinct partner organizations. Many of the participants were involved in the Define phase and prior research activities in Regions 7 and 8, which helped to ensure continuity and the application of learnings from the Define phase. Upon the completion of the workshop, the goal was to have a set of initial ideas represented as simple, low-fidelity prototypes that could be taken out to the regions to be rapidly tested and refined with representatives from local communities, mining camps, and health facilities.

The Imagine Workshop began with a recap presentation of the 11 insights that emerged from the Define phase. As each insight was presented, workshop participants were asked to consider:

- What is new or interesting?
- What are the initial ideas that come to mind?

Objectives of the Imagine Workshop include:

- Review and refresh insights from Define stage
- Immerse the workshop group in the research findings
- Move into “idea generation” frame of mind

##### Use of HMWs and development of prototypes

During the workshop, participants were divided into five groups. Each group was given two insights and corresponding design challenges framed as HMW questions that were previously developed. These questions were used to guide the generation of new ideas for possible interventions. In all, more than 790 ideas were generated and then later collapsed into eight broad categories. From the vast set of potential solutions, the Breakthrough ACTION team examined trends and themes and clustered similar ideas into refined groups of concepts. The concepts identified were then distributed to the groups of workshop participants who further developed the ideas by merging smaller ideas and adding details where necessary.

Once a final idea was decided on for each theme, workshop participants were tasked with building tangible, low-fidelity versions of the ideas, known as prototypes. Prototypes allowed the team to test the idea with real audiences quickly and cheaply.

A prototype might take many different forms. It is important to note that the prototype might look quite different to what the idea would look like in reality, if it were implemented. A prototype is useful as long it allows us to learn about the idea by proving or disproving our assumptions.

Low-fidelity prototypes were constructed from craft materials such as Play-Doh, colored paper, markers, and string. Some used digital mockups and photoshopped images.

#### Design & test Stage II: prototype and test

##### Prototyping and field testing methodology

Two teams using a similar composition to the research teams during the Define phase returned to Regions 7 and 8 for prototype testing in March 2019. The interventions were tested with a total of 145 persons including miners (56), malaria testers [20], camp managers [15], community members (32), health workers [15], regional administrative officials [5], and others [2]. On each day of testing, users were given the opportunity to interact with the prototypes and provide feedback on the ideas. Inquiries and observations were made about their initial thoughts and reactions to the interventions; what they liked and didn’t like about each one; whether they thought the intervention was useful; how each intervention could be improved, and any other suggestions they wanted to share. Based on the feedback received, prototypes were either refined, discontinued, or new ideas generated. Throughout the process, researchers followed two prototyping principles: [1] the greatest value should be created for the user with the smallest input of resources, and [2] prototypes should be put in the hands of the user (for testing) as quickly as possible.

##### Decision-making process

The project team aimed to identify the most promising ideas that could improve malaria testing and treatment-seeking behavior. Over the course of one week, the two teams adhered to the following methodology:

- Refine: Develop the ideas into something we can build by identifying assumptions and designing the finer details of the concept.
- Prototype: Build ideas into tangible low-fidelity models that can be physically taken and tested within communities.
- Test (Monitor & Evaluate): Give users the chance to interact with the prototypes and provide feedback on the idea. At this stage, some ideas will possibly be identified as undesirable, infeasible, or inappropriate, and will be discontinued.
- Imagine: Generate new ideas based on new findings and results of testing our assumptions. Reflect on what was learned the previous day, and decide how that will affect the ideas in development.

This methodology was continuous to give space for iteration of the prototypes with the target audience throughout the week.

## Results

### Define phase

**Fig. 3:**
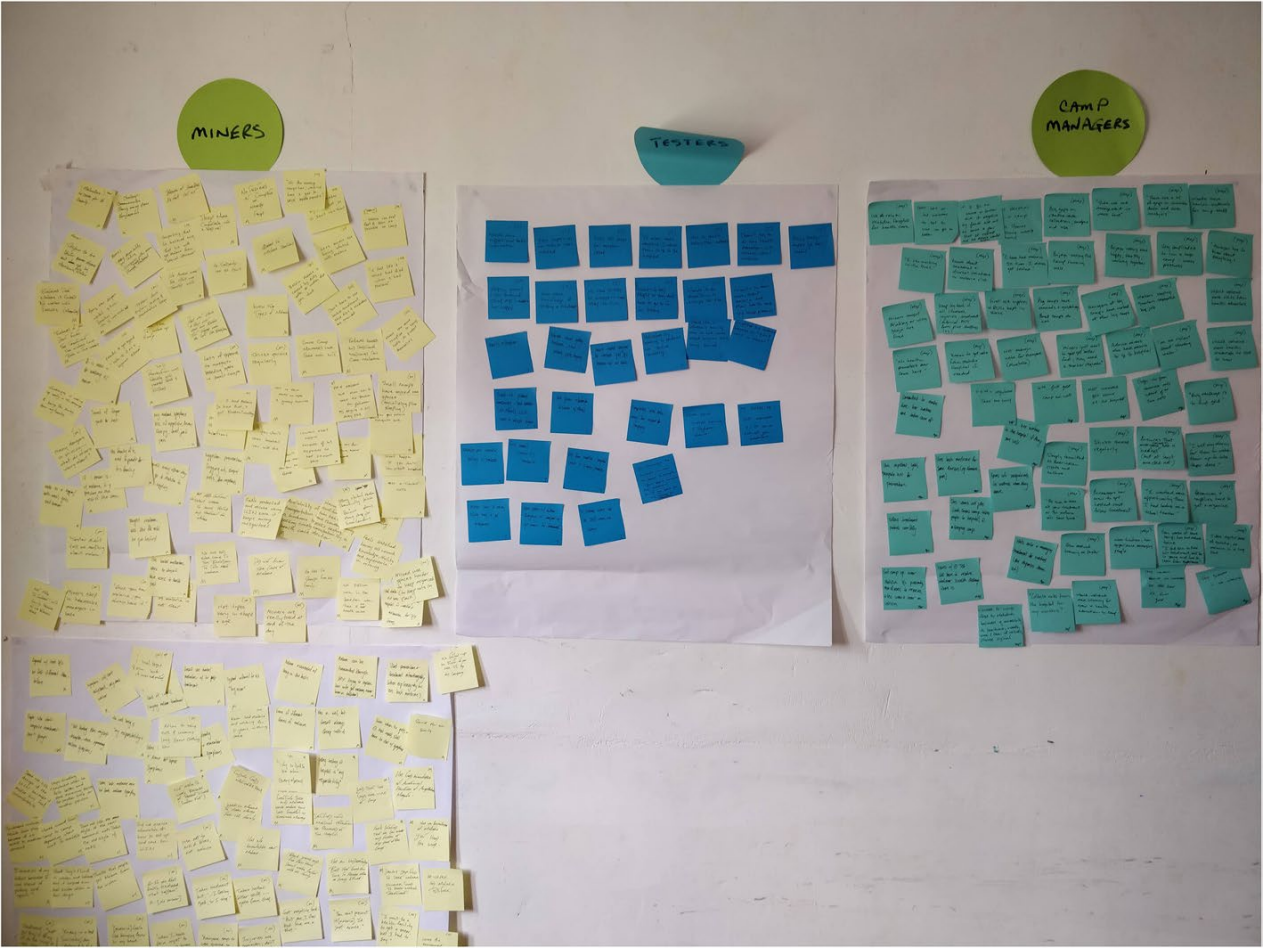
Insights harvesting of field research

Following in-depth interviews and FGDs with more than 100 miners, camp owners, testers, MoH officials, and other stakeholders, the data gathered in the field was synthesized into 11 insights or key findings that helped the team better understand attitudes, beliefs, and behaviors related to malaria testing and treatment. Figure 3 demonstrates the insights harvesting process. These insights, outlined in Table 2, were later used to inform the Design & Test phase of the HCD process and served as a framework for identifying solutions for barriers to the intended behavior.

**Table 2:** Outline of major insights from the Define phase

Theme	Insight	Quote
Risk perception	Malaria is seen as routine and commonplace; it is not considered a major health risk for many communities.	“If you want to prevent malaria—don’t come to the bush.”—Miner
Malaria knowledge and preventive behaviors	There are many contradictions between what people know about malaria and how they behave.	“Once you have malaria, you always have it.”—Miner
Adherence and non-adherence to correct treatment	Undesirable medication side effects cause some miners to stop treatment as soon as they feel better, while the need to get back to work and be able to keep working causes other miners to follow the regimen.	“I feeling good, so I stop [taking malaria treatment].”—Miner
Traditional and self-treatment for malaria	Commonly accepted practical solutions to diagnose and treat malaria, which differ from official recommendations, are often preferred due to convenience and personal experience with these treatments.	“I use herbal treatments for malaria if there is no access to a health facility.”—Miner
Testing	The role of volunteer testers in providing free malaria testing and treatment services is not fully known, understood, or appreciated by miners and other clients.	“I didn’t realize that testing and treatment is offered for free.”—Miner
Job motivation	Miners and camp workers often prioritize financial/economic gain over their health concerns.	“Making money is my first priority.”—Camp Manager
Mining camp environment	Miners and their camp managers have strong and respectful relationships because they need each other to be successful at their jobs.	“During work or at the landing you gotta look out for each other because we’re from the same country.”—Miner
Health care sources	Health facilities are a desired option for health care services, but people will access other sources, if necessary, due to transportation, time, distance, and cost constraints.	“Best thing is to go to the hospital.”—Camp Manager
RDT training	The RDT training provided by the MoH is effective; however, testers would like to be trained to provide additional health services.	“I didn’t know how malaria was spread until I sat in on RDT training.”—Tester
Communication	Health communication and health promotion activities and materials, including radio programs, exist but are undeveloped and underutilized.	“More public awareness is needed about testing in remote areas.”—Radio Broadcaster
Coordination and communication gaps	A lack of coordination and communication between stakeholder groups reduces the effectiveness of the National Malaria Programme.	“Reporting and feedback mechanisms are lacking between testers and regional teams.”—Tester

### Design & test: Stage I and Stage II

During the Imagine Workshop, participants produced a total of 792 ideas which were grouped into eight major themes, refined into workable concepts, then prototyped.

1. The idea of a *SBC campaign* started off with four distinct creative concepts, each with a campaign name, slogan, and simple accompanying visual(s) to test. “Go for Gold” was meant to show the economic impact of not using “gold standard” MoH-approved tests and treatment. “Man vs. Mosquito” pitted humans against mosquitoes in a humorous, comic-based approach. “Little Mosquito, Big Problem” aimed to increase malaria risk perception by appealing to both the head and heart through statistics and testimonials. “Don’t Give Me Malaria**,** Man” focused on the human’s role in malaria transmission and the impact it could have on the entire camp/community.
2. *Malaria trainer support materials* were developed as a job aid for volunteer testers to provide basic information on malaria to miners and to improve the accuracy and quality of the service provided. Originally, the tool was designed in the form of a flipchart, one side with a picture depicting relevant information about malaria for the miner and the other side with text to help the tester explain the visual.
3. A comprehensive *RDT branding* strategy was created to increase the visibility of and trustworthiness in MoH-approved testers, services, and products. It included a consistent visual element to convey the availability of free malaria testing and treatment services.
4. *Standardization of the RDT program* was a partnership between MoH and private importers of malaria supplies intended to standardize RDT kits and treatment supplies to increase the accuracy of malaria test results since WHO prequalified RDT kits and treatment that are proven to be highly accurate and effective are preferred compared to other available options.
5. The *treatment adherence* idea started as a simple pill box container that split daily doses into separate compartments and included additional information such as when to take each dose. See Fig. 4 for an initial prototype of the treatment adherence process.

**Fig. 4:**
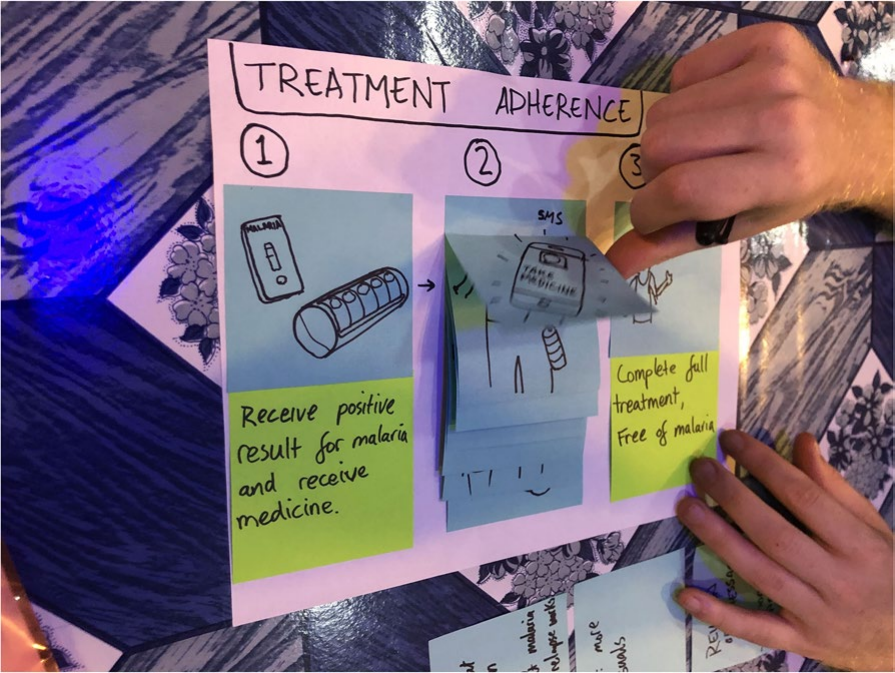
Prototype of treatment adherence steps
6. *Collaboration with private sector transportation* was envisaged as a coordinated network of transportation companies (e.g., boat, plane, car) to facilitate movement of supplies and reports to and from camps to minimize stock-outs, enhance reporting, and reduce non-reimbursable out-of-pocket expenses from local/volunteer staff.
7. *MalaApp* was a phone app designed to improve the ease, speed, and quality of reporting; improve testing and treatment services; and reduce stock-outs. The prototyped app was meant to help testers easily and accurately fill out necessary documentation, report stock-outs, request supplies, and contact other testers. The app also included educational information on malaria transmission, symptoms, how to use LLINs, and the recommended malaria treatment. Figure 5 demonstrates a low-fidelity mockup of the app screens.

**Fig. 5:**
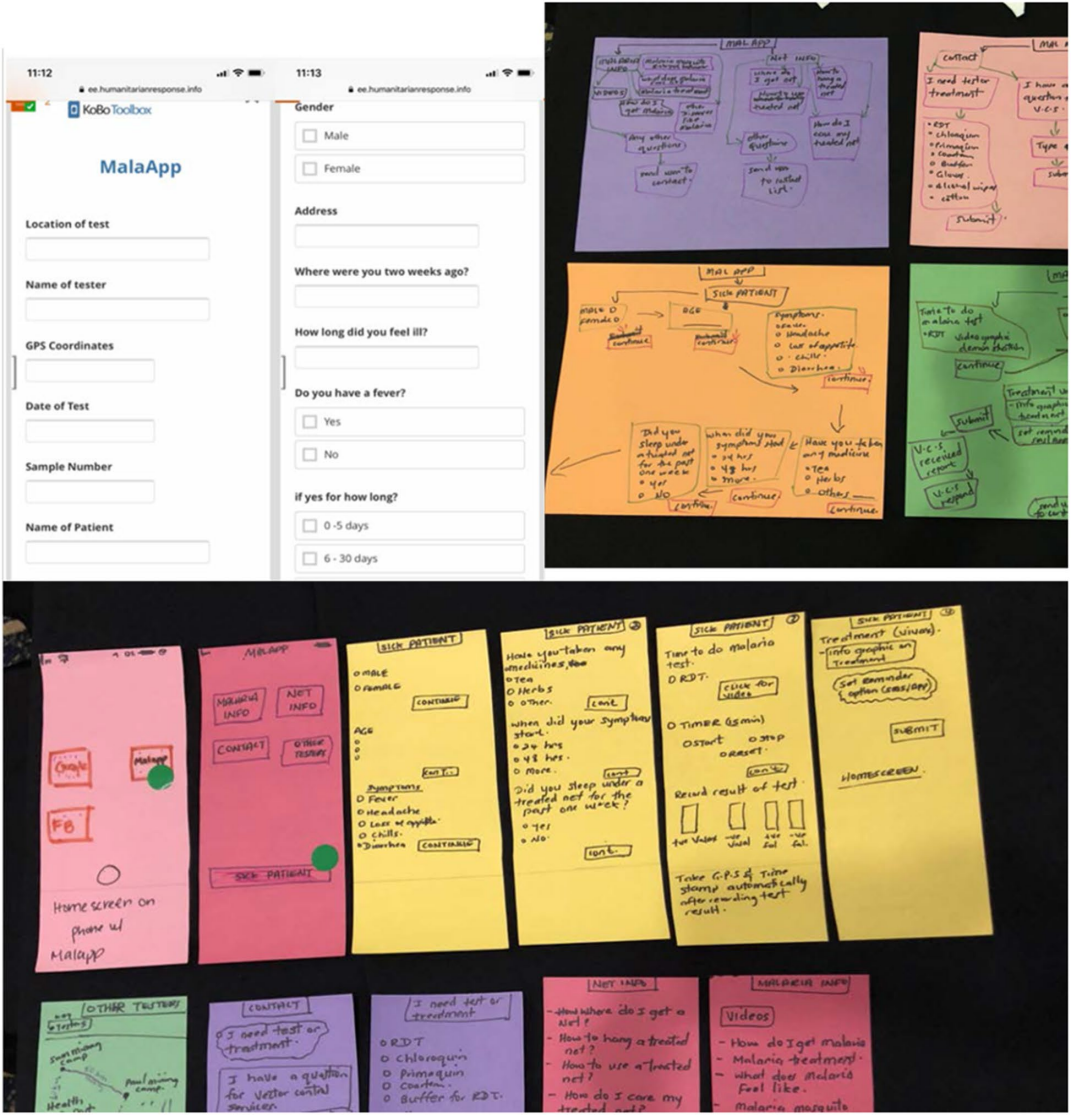
Low-fidelity prototype of the MalaApp
8. The **Malaria Fighters Training of Trainers network** was an updated structure for the RDT program, with current testers serving as trainers and supervisors for new testers, building a network in which the health workers would support themselves, decentralizing training and increasing the number of active testers.

### Final prioritized solutions

**Fig. 6:**
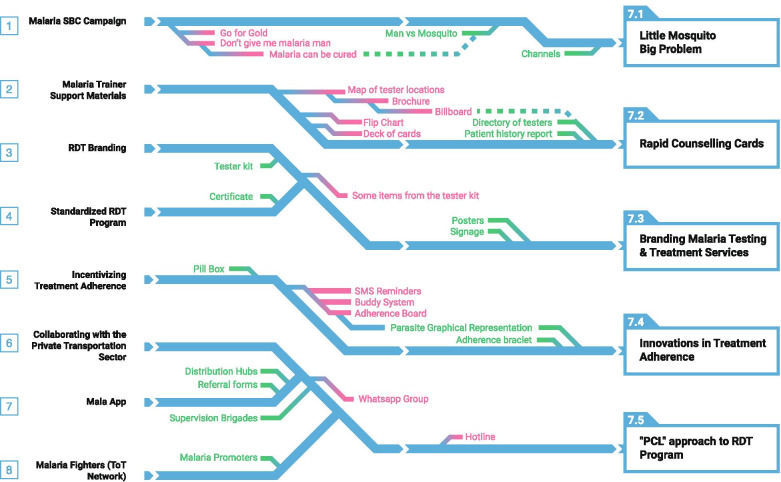
Prototype progression throughout field testing

Rapidly building and testing tangible, low-fidelity versions of each concept provided valuable, early user feedback that helped to refine or abandon design ideas. Field testing of eight prototypes yielded five design prototypes that emerged as the most desirable (Fig. 6). The five prototypes were further reviewed and refined into the following solutions:

1. The “**Little Mosquito, Big Problem” (LMBP)** creative concept was most preferred by miners for the SBC campaign since it was easily understood, humorous, and captured their attention best (Fig. 7). The “Don’t Give Me Malaria, Man” concept, which examined the human element of transmission, was found to be stigmatizing by some and the “Go for Gold” concept was confusing to miners as they did not understand the intended economic angle.

**Fig. 7:**
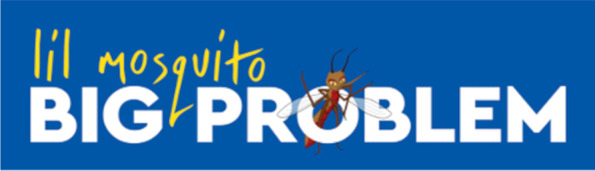
Social behavior change slogan to address malaria risk perceptions
2. The LMBP campaign will address four major topics: 1) malaria risk perception, 2) testing for malaria within 24 h of experiencing symptoms, 3) treatment adherence, and 4) LLIN use. It will include a soca song (style of Caribbean dance music) and animated music video, radio spots, an animated video mini-series, a social media personality, and posters. Initial feedback from miners on a LMBP campaign was: “It’s reality - the mosquito is so little, but it is a big problem”; “This is a good message. You’re showing them the facts”;
3. Testers preferred the **Rapid Counseling Cards (RCC)**—a stack of color-coded counseling cards with a checklist that would allow them to counsel clients only on topics relevant for their needs (Fig. 8). Testers felt the job aid in the form of a flipchart was too cumbersome for their busy schedules so the RCC was designed smaller and with the accompanying checklist to tailor the counseling to each client. The counseling cards have an image on the front and pertinent information about malaria prevention, transmission, and treatment on the back. The counseling checklist also represented an attempt to collect more information about the content of discussions between testers and clients that could in turn be shared with VCS staff and used to develop effective interpersonal communication strategies for testers. Some feedback from testers on the RCC included: “This is good information for everyone to know”; “I would go through the entire thing while they wait for the test results”; “This will be good for me, everything is really good.”

**Fig. 8:**
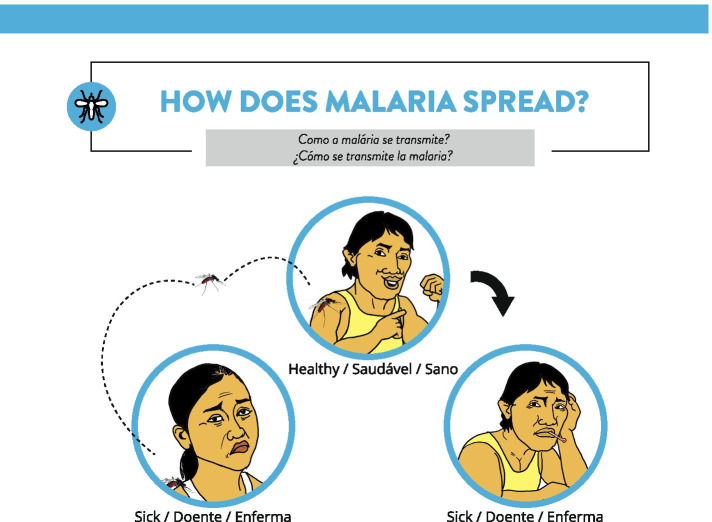
Example rapid counseling card explaining malaria transmission
4. The **branding of malaria testing and treatment services** with the use of a flag intended to raise the visibility of free, MoH-approved malaria testing and treatment services under the Ministry’s RDT program was widely appreciated by testers (Fig. 9). The branding package also includes a tester’s toolkit and certificate of completion of the RDT training. The toolkit will include the rapid counseling cards, a laminated card with the treatment regimen for different types of malaria, referral forms to be used in cases of stock-outs, and a flashlight to help testers read cartridges in the evening. It will also contain malaria test kits, treatment, gloves, and reporting forms provided by VCS. Miners thought that branding malaria testing and treatment services would be useful for them:” If I know where they have free test I wouldn’t pay for the other one”; “I didn’t know there was free test here. Now I know. I will come.” Testers also shared their feedback on a branded toolkit: “This [box] would be good to keep things safe from the heat”; “I would frame [the certificate] and keep it on my wall so when they come, they can see it”; “The flashlight is a great thing because sometimes when you’re in here, you don’t get to see the cassette properly.”

**Fig. 9:**
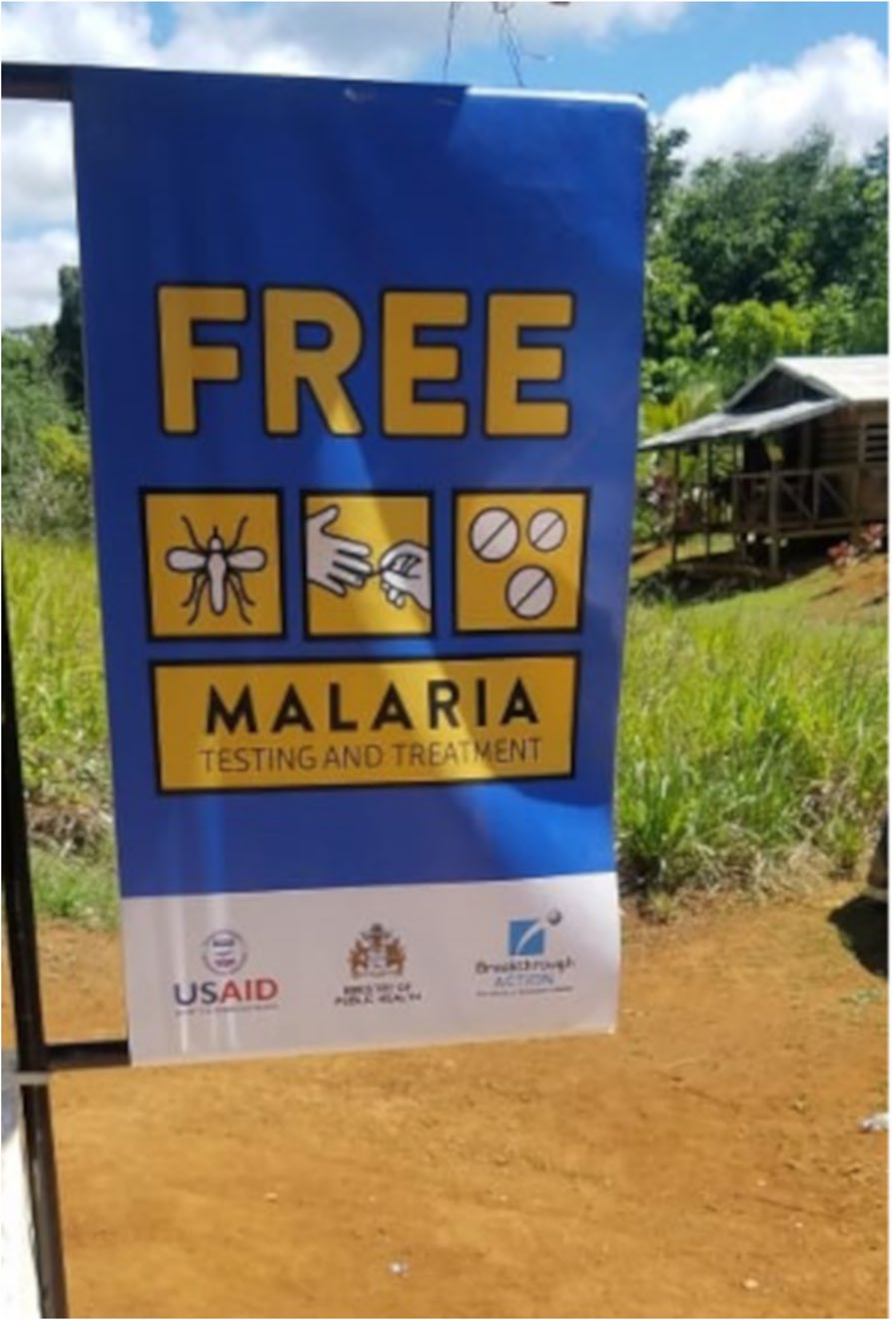
Example sign posted outside malaria testing and treatment center
5. The two products, collectively known as **innovations in treatment adherence**, work together to simplify treatment, provide treatment reminders, and encourage treatment completion (Fig. 10). Pill cases to improve adherence were found to be bulky. Miners preferred a lighter and more compact tablet strip that separates daily prescribed dosages in individual packets. Each packet would also include a visual representation of how parasites in the body are decreased each day the treatment is taken. A second product—a wristband that uses an audible reminder to indicate when the user should take their treatment—would accompany the tablet strip. Feedback on this concept included: “People might realize how important each pill is for them”; “It makes it clearer what to take when”; “This would show I’ve been taking it for three days, but I still have a lot of parasites left”; “This would be good, should make it with brighter colors so everybody is accountable for the person”; “I would use it to remind me when I’m out there.”

**Fig. 10:**
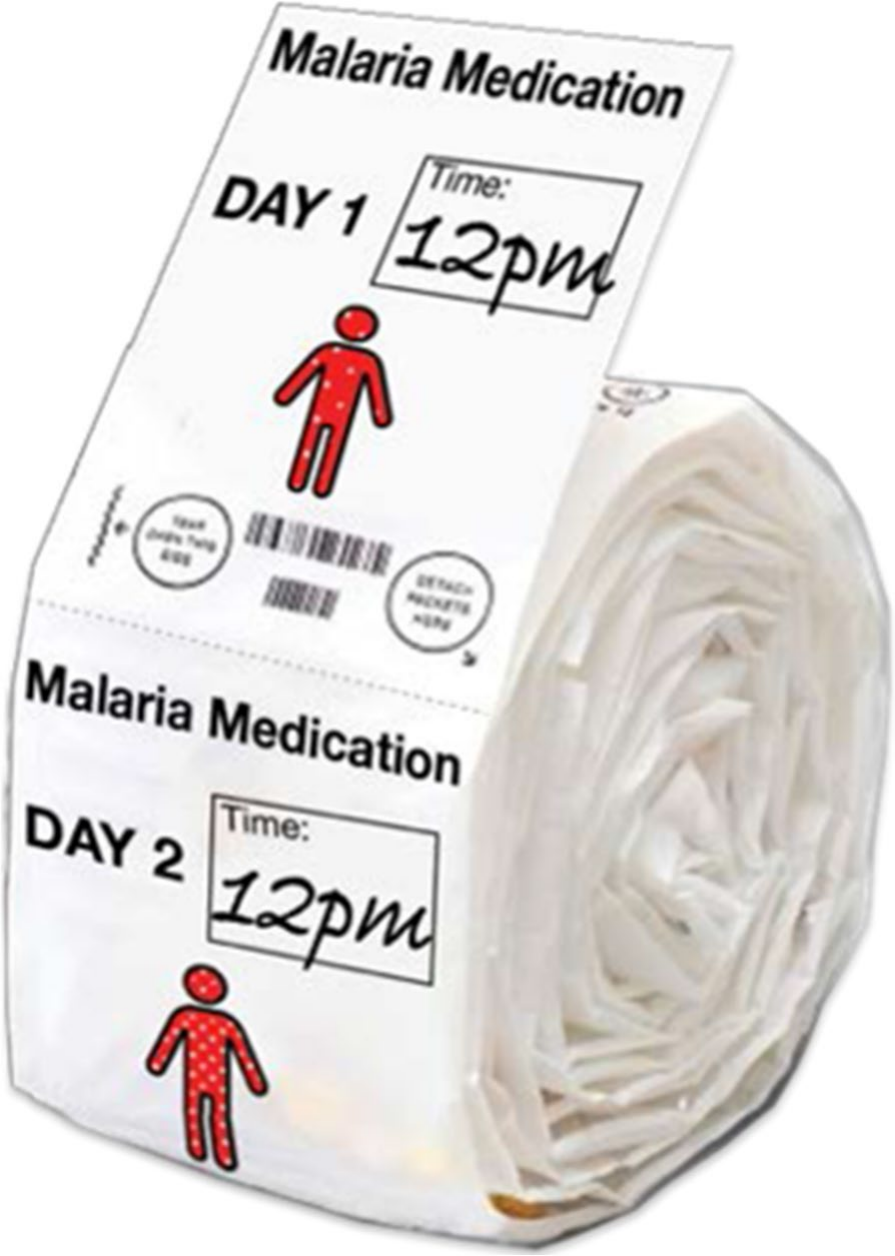
Prototype of daily packaged pill medication
6. Improvements to the current RDT program—the *Participants, Content, and Logistics (PCL) approach*—was favored over the MalaApp and Malaria Fighters. The PCL approach would address the scaling up of testers, antimalarials, and other malarial commodities in difficult-to-access mining areas. It includes hubs for distribution, reporting, medical supplies, and training facilitated by a mobile brigade. RDT testers will be supported by implementing a referral form that caters for stock-outs and cases that testers are not equipped to manage. Experiences shared by testers that inspired this solution were: “I went to the hospital for supplies and they didn’t have any. I never came back”; “I asked for materials from people around, but nobody shares.”

Breakthrough ACTION Guyana piloted the RCCs and counseling checklist, branding for malaria testing and treatment services and small components of treatment adherence (treatment adherence handouts), and PCL approach (referral forms) in Regions 7 and 8 over a three-month period (June–August 2019). In each pilot area, volunteers were trained to become new RDT testers while active testers received a refresher training. During the trainings, testers were oriented on how to use the rapid counseling cards and counseling checklist, branding materials, treatment adherence handouts, and referral forms. Immediately after the orientation, FGDs were conducted to elicit feedback from trainers on the materials. Intermittent supervisory visits were conducted by Breakthrough ACTION and VCS during the pilot period to monitor the use of the materials, collect completed forms and checklists, and coach testers on the use of the new products and practices.

## Discussion

This article highlights the use of HCD to understand challenges and opportunities to encourage gold miners to seek malaria testing and treatment services, and potential solutions which could be implemented. Kim et al. used HCD to understand insights around consumer preferences for LLINs in Ghana to improve the uptake of LLINs [8]. Through the use of HCD methodologies, the authors understood that preference of LLINs were linked to convenience, comfort, and aesthetics. Sikombe et al. used HCD to engage with 900 community stakeholders to inform and encourage buy-in for implementation of Zambia’s National Malaria Elimination Program (9). This article contributes to additional research and application of HCD on uptake of malaria health services by describing the process and results of a HCD-driven process to improve malaria testing and treatment outcomes.

In a separate article, the Define phase qualitative research activities were analyzed according to the Integrated Behavior Model, which offers a structured framework to understand attitude, perceived norms, and personal agency factors that influence behavior [12, 17]. The synthesis of the qualitative field research data yielded similar reasons to explain existing malaria testing and treatment-seeking behavior, using the Integrated Behavior Model (a social-behavioral science approach) and Insights Harvesting and Validation (a design approach)—the method described in this manuscript. Both analysis methods demonstrated that enabling factors for malaria testing and treatment seeking behavior were geographic proximity and internal motivation to seek and/or complete testing and treatment services offered by the MoH. Miners who used informal treatment or stopped MoH treatment early prioritized feeling better as soon as possible.

The benefit of using HCD to improve malaria outcomes can be seen in the numerous ideas generated with perspectives across the public health and gold mining communities. In Guyana, the use of HCD among gold miners in the hinterlands generated concepts beyond traditional communication solutions such as the innovations in treatment adherence, which include repackaging the medication and reminder wristbands to ensure treatment adherence. Additionally, the PCL approach aims to consider larger systemic change to malaria infrastructure, both in the Guyanese hinterlands and centrally. Communication campaigns and counseling cards were seen and used in previous malaria-based initiatives. In reference to the “Little Mosquito, Big Problems” campaign, health communication interventions have been demonstrated to improve malaria prevention and treatment outcomes within African countries [18, 19]. Rapid counseling cards have been used in other health areas such as responsive feeding counseling for families in Ghana and nutrition behavior change programs in Zambia with promising results [20].

The HCD process of rapid iteration of low-fidelity prototypes and co-creation with the target audience allowed the project team to quickly apply learnings to improve, discontinue, or generate new prototypes. During field testing an additional insight was discovered—that many miners believed that once infected, malaria remains in the body and “raises up” when immunity is low. The belief was expressed generally for all types of malaria and was not specific to *Plasmodium vivax*. This insight was then utilized to enhance the idea of repackaging medication, by adding a visual on the packet of decreased parasitemia as the medication is taken. Additionally, due to field testing, potentially costly mistakes were avoided, like the implementation of the MalaApp, which the vast majority of volunteer testers felt could not work in their setting because of phone and internet connectivity issues. Ideas that were discontinued also provided valuable insights about the target audience. For example, miners interpreted the “Go for Gold” campaign slogan literally rather than the malaria messaging and did not understand text-based materials due to literacy challenges; therefore, solutions had to be highly visual and direct.

Overall, public health interventions should be evidence-based. This means that, to the extent possible, when HCD is used for intervention design it should try to adhere to appropriate standards for scientific rigor like any robust qualitative research approach, while also trying to avoid biases about stakeholder experience and maintaining an open-minded agnosticism about the potential solutions to a design challenge. Some of the most essential principles of the scientific method are falsifiability, replicability, and generalizability [21, 22].

Falsifiability refers to whether or not expectations or questions—in this case, about malaria testing and treatment—are stated in a way that could potentially be disproven through observation [23, 24]. Statements or expectations that cannot be disproven (e.g., “getting malaria is inevitable,” cannot be proven or disproven because the future cannot be known with certainty) are not stated in a scientific way. In scientific research, questions typically take the form of hypotheses or predictions. In HCD, the following are types of hypotheses: formation of questions for the of Lines of Inquiry, the framing of HWMs, and the generation of potential solutions through iterative ideation. The project team therefore exercised caution to ensure that any question raised must be potentially testable through observation.

Replicability refers to whether others outside the project research team, using the same approach and methods, would reach the same conclusions or results [23]. If not, this suggests that there is either a flaw in the approach/method by which information is gathered or there is bias in the analysis, or both. For example, would others using the same approach produce the same or similar prototypes for testing? The open-ended, “beginner’s mind” perspective often produces surprising and unexpected insights, but effort is required (e.g., through collective processing of Discovery date, prototype testing, and stakeholder consultation) to guarantee that results are grounded in reality and are not purely subjective. Throughout the Define and Design & Test phase, the project team engaged with key stakeholders and gold miners to keep insights and potential solutions grounded.

Generalizability refers to the extent to which conclusions/results are locally and contextually specific or could apply to other contexts, as well [25]. If there is low generalizability, chances of taking an intervention to scale are limited. HCD typically aims to produce solutions that meet the specific needs of a stakeholder group(s) in a particular place and time. While it attempts to be solution-agnostic and sensitive to local stakeholder needs, the HCD process must be designed and implemented to produce evidence that gives us confidence those solutions will work in the larger population-at-risk. The project teams attempted to prioritize solutions that could work across regions, but whether solutions are generalizable will be clearer in the Apply phase. Further, in order to implement the solutions in similar mining and logging settings like the Guyana Shield, a pilot of local adaptions would be warranted; however, there may not be need to repeat the entire HCD process.

Practical challenges raised by adherence to these principles include ensuring that interviewers and observers with little or no formal training in qualitative research methods understand and can use the research methods effectively. But limited time and resources for training in the present program required real-time learning by the research team in the field. The teams had to be flexible enough to pause for reflection and to allow for additional mentoring and practice on the job for team members who were new to this kind of work. Some members of each team became more adept at interviewing than others, so teams adjusted the data collection roles accordingly.

A second challenge arises from the fact that much of the analysis and interpretation of discovery stage data is done collectively and—sometimes—intuitively, rather than as part of a formal hypothesis-testing approach. Therefore, the research team aimed to document the process in as much detail as possible in order to permit retrospective review and validation of the insights that are achieved and the decisions that are made.

A third challenge revolves around the contrast between the HCD goal of developing prototype solutions that are locally appropriate and feasible at the time and place when the research is conducted, and the need to develop interventions that are likely to work over time, or even to be sustainable in the long term and scalable in comparable settings. Judgments about the sustainability of potential solutions have to be “crowdsourced” in the sense that the relatively short HCD discovery-design-pilot test process does not typically include longer-term evaluation of intervention impact. So instead, the HCD process must rely more on local wisdom from stakeholders and the experience of experts to judge which solutions are most likely to have longevity in practice. Efforts were required to validate prototype solutions through pre-testing in each of the regions as intervention activities were rolled out. In the Apply phase, the project team will test for solution feasibility and sustainability in partnership with key stakeholders for the project (e.g., mining organizations, MoH, PAHO/WHO, and USAID).

For Breakthrough ACTION Guyana’s specific focus on testing and treatment adherence, the introduction of HCD approaches was invaluable. Prior to this project, relatively little contextual knowledge was available about the populations at risk in the remote mining regions of Guyana, even among local health experts. HCD helped to draw back the curtain and dramatically expand understanding of the complex situational dynamics of malaria transmission and prevention through the elicitation and analysis of stakeholder experiential narratives. For the stages documented here, the team focused on generating a large volume of ideas; the Apply phase focuses on the evaluation, implementation, and scale of promising interventions.

There are implementation concerns and limitations of the HCD approach, which in turn became advantages. First, while the user-centered focus is one of the key strengths of HCD, this often necessitates that the project team have basic research skills. The additional days dedicated to research training for the discovery and design teams was essential to standardize the HCD process, determining the replicability and generalizability of outcomes. Secondly, the iterative learning inherent to HCD can also lengthen project timelines. It has often been said that the use of HCD allows projects to “fail fast”—learning quickly what does and doesn’t work—then make adjustments to prototypes before taking solutions to scale. The need to validate and confirm design decisions often requires multiple rounds of prototype testing under different conditions and with varying stakeholder groups. This adherence to higher scientific standards can take longer to implement than projects may expect. Although in practice HCD may not necessarily produce actionable and effective solutions at scale more quickly than other common approaches, user-centered approaches and design thinking are still valuable in that pursuit.

Finally, common HCD models and approaches tend to stop short of population-based implementation and evaluation of impact at scale. While the use of qualitative methods in program evaluation are important for understanding stakeholder response to an intervention, quantitative methods using structured questionnaires and population-based sampling of respondents are essential for measuring the magnitude of impact. If HCD is to become an integral part of larger-scale health and development initiatives, it needs to be integrated with other behavioral science research approaches that help complete the picture of what works and why. Following the SBC Flow Chart, the project team plans to implement the finalized solutions at scale across Regions 1, 7, and 8 with accompanying population-based baseline and endline evaluations. This may help address some of the earlier mentioned weaknesses of the HCD approach. At the time of writing the paper, the project had initiated the Apply phase.

## Conclusion

In order to encourage Guyanese hinterland-based gold miners to seek malaria testing and treatment services, the project team used HCD to design, develop, and implement solutions with local partners. The project team synthesized 108 qualitative interviews into 11 insights on risk perception, malaria knowledge, preventive behaviors, traditional and self-treatment, adherence to the correct treatment, testing, and coordination and communication gaps. Of the 792 ideas generated from 33 “How might we…?” questions, eight emergent concepts were prototyped and refined in the field with 145 miners, camp managers, and stakeholders into the final five prototypes. The final prototypes were the following: “Little Mosquito, Big Problem” social behavior change campaign; rapid counseling cards; branding malaria testing and treatment services; innovations in treatment adherence; and a PCL approach. These prototypes span product-, service-, and systems-level approaches, which demonstrates the multi-faceted nature of the challenge. Using HCD within public health poses unique challenges, especially around the expectation that public interventions meet falsifiability, replicability, and generalizability principles, but also allows for more meaningful work with beneficiarious and key stakeholders to reveal and understand the public health problem and identify solutions that may work.

## Data Availability

The datasets used and/or analysed during the current study available from the corresponding author on reasonable request.

## Abbreviations

FDG: Focus group discussions
GFATM: Global Fund to Fight AIDS Tuberculosis and Malaria
HCD: Human-centered design
HMW: How might we
LLIN: Long lasting insecticide treated nets
LMBP: Little Mosquito Big Problem
MoH: Ministry of Health
NMP: National Malaria Programme
PAHO/WHO: Pan American Health Organization/World Health Organization
PR/HPU: Public Relations/Health Promotions Unit
PCL: Participants, contents, and logistics
RCC: Rapid counseling cards
RDT: Rapid diagnostic test
SBC: Social and behavior change
SBCC: Social and behavior change communication
USAID: United States Agency for International Development
VCS: Vector Control Services
